# Hospital admission at the time of a postpartum psychiatric emergency department visit: the influence of the social determinants of health

**DOI:** 10.1017/S2045796021000238

**Published:** 2021-04-23

**Authors:** Lucy C. Barker, Susan E. Bronskill, Hilary K. Brown, Paul Kurdyak, Simone N. Vigod

**Affiliations:** 1 Department of Psychiatry, University of Toronto, Toronto, Canada; 2 Institute of Health Policy, Management and Evaluation, University of Toronto, Toronto, Canada; 3 ICES, Toronto, Canada; 4 Women's College Hospital, Toronto, Canada; 5 Department of Health & Society, University of Toronto Scarborough, Toronto, Canada; 6 Centre for Addiction and Mental Health, Toronto, Canada

**Keywords:** Psychiatric Services, Postpartum Mental Health, Emergency Psychiatry

## Abstract

**Aims:**

Social determinants of health have the potential to influence mental health and addictions-related emergency department (ED) visits and the likelihood of admission to hospital. We aimed to determine how social determinants of health, individually and in combination, relate to the likelihood of hospital admission at the time of postpartum psychiatric ED visits.

**Methods:**

Among 10 702 postpartum individuals (female based on health card) presenting to the ED for a psychiatric reason in Ontario, Canada (2008–2017), we evaluated the relation between six social determinants of health (age, neighbourhood quintile [Q, Q1 = lowest, Q5 = highest], rurality, immigrant category, Chinese or South Asian ethnicity and neighbourhood ethnic diversity) and the likelihood of hospital admission from the ED. Poisson regression models generated relative risks (RR, 95% CI) of admission for each social determinant, crude and adjusted for clinical severity (diagnosis and acuity) and other potential confounders. Generalised estimating equations were used to explore additive interaction to understand whether the likelihood of admission depended on intersections of social determinants of health.

**Results:**

In total, 16.0% (*n* = 1715) were admitted to hospital from the ED. Being young (age 19 or less *v*. 40 or more: RR 0.60, 95% CI 0.45–0.82), rural-dwelling (*v*. urban-dwelling: RR 0.75, 95% CI 0.62–0.91) and low-income (Q1 *v*. Q5: RR 0.81, 95% CI 0.66–0.98) were each associated with a lower likelihood of admission. Being an immigrant (non-refugee immigrant *v*. Canadian-born/long-term resident: RR 1.29, 95% CI 1.06–1.56), of Chinese ethnicity (*v*. non-Chinese/South Asian ethnicity: RR 1.88, 95% CI 1.42–2.49); and living in the most *v*. least ethnically diverse neighbourhoods (RR 1.24, 95% CI 1.01–1.53) were associated with a higher likelihood of admission. Only Chinese ethnicity remained significant in the fully-adjusted model (aRR 1.49, 95% CI 1.24–1.80). Additive interactions were non-significant.

**Conclusions:**

For the most part, whether a postpartum ED visit resulted in admission from the ED depended primarily on the clinical severity of presentation, not on individual or intersecting social determinants of health. Being of Chinese ethnicity did increase the likelihood of admission independent of clinical severity and other measured factors; the reasons for this warrant further exploration.

## Introduction

Psychiatric disorders are among the most common complications of childbirth (O'Hara and Wisner, 2014). While most psychiatric presentations can be treated in an outpatient setting, about 1% of postpartum individuals (women and childbearing individuals of other gender identities) present to an emergency department (ED) for a psychiatric reason in the first year after delivery (Barker *et al*., 2016). This high-risk group comprises the most severe psychiatric presentations, with serious potential for negative maternal and child health implications outcomes (Luykx *et al*., 2019). Among all postpartum ED presentations, psychiatric disorders are the most likely to lead to admission (Batra *et al*., 2017); admission occurs for approximately one in seven individuals with postpartum psychiatric ED presentations (Barker *et al*., 2016). Yet, little is known about what contributes to admission decisions. Ideally, the decision to admit a postpartum individual to hospital upon presentation to the ED for psychiatric reasons would primarily be based on clinical presentation, with maternal and infant safety being paramount. It is also important to ensure that admissions do not occur unnecessarily, and decisions need to consider the potential impacts of disruptions to infant care (e.g. breastfeeding) and maternal–infant bonding during admissions.

Social determinants of health such as age, ethnicity, gender and income, shaped by socio-political contexts, can impact health outcomes and drive health inequities (Solar and Irwin, 2010). In non-perinatal psychiatric populations, a person's likelihood of being admitted to hospital from the ED can be influenced by social determinants of health. Sometimes, this can be necessary. For example, individuals living in poverty are more likely to be admitted when precarious housing or inadequate social services are identified as barriers to successful outpatient management (Brooker *et al*., 2007; Kroll *et al*., 2018). Sometimes homeless individuals are presumed to be presenting to the ED to only meet sustenance needs (e.g. obtain shelter), reducing the likelihood of admission (Unick *et al*., 2011). Social determinants may also intersect with each other in this context. For example, in an American study, being employed increased the likelihood of psychiatric admission from the ED for white individuals, but not for those of Asian backgrounds (Unick *et al*., 2011). Understanding whether and how social determinants of health impact admission decisions is important to identify potential biases and ensure equitable care.

Whether social determinants of health impact hospital admission decisions for postpartum individuals with mental illness is unknown. Social determinants of health, individually and in combination, are known to influence the development of postpartum mental illness, access to postpartum mental health care and the experience of this care. Those who are younger, experiencing poverty, minoritised and who are immigrants and refugees are at elevated risk for postpartum mental illness, for facing barriers in access to care and for having negative experiences of care related to historical and ongoing patterns of systemic discrimination (Kurtz Landy *et al*., 2008; Hodgkinson *et al*., 2014; Barker *et al*., 2016; Vigod *et al*., 2016; Faulkner *et al*., 2019; Watson *et al*., 2019). If social determinants of health impact whether a postpartum individual is admitted to hospital from the ED independent of clinical need, this may signal bias in admission decisions and a need for interventions to ensure equity in likelihood to be admitted. A greater understanding of this impact could help guide more equitable decision-making and improve health outcomes in this at-risk population.

Using population-based data from Ontario, Canada, this study explored the association between six social determinants of health (age, income, immigration, region of residence, ethnicity and neighbourhood diversity) and the likelihood of hospital admission at the time of a postpartum psychiatric ED visit. We hypothesised that each social determinant would impact the likelihood of admission, and that the concurrent experience of multiple forms of marginalisation would have synergistic effects on an individual's likelihood of admission.

## Methods

### Conceptual framework

**Fig. 1.**
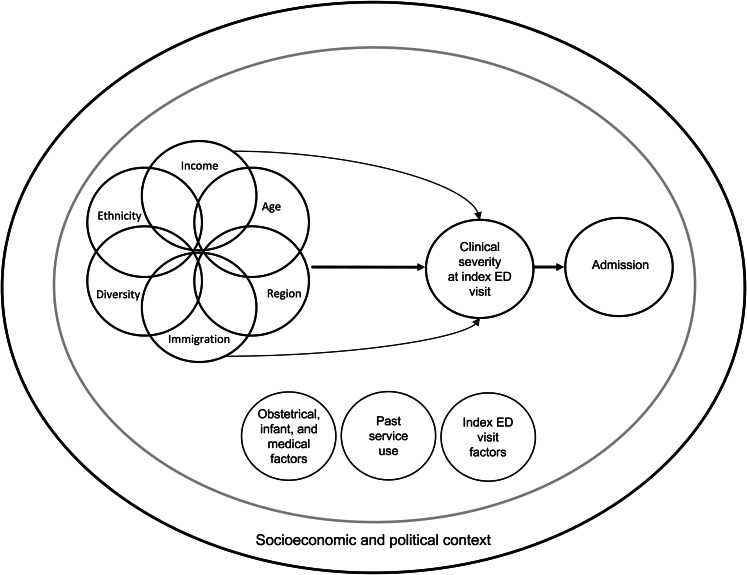
Conceptual framework.

This study used an intersectional framework to understand the relation between social determinants of health, individually and in combination, and psychiatric hospital admission (Fig. 1). Intersectionality, originating from Black feminist scholarship, elucidates the impacts of concurrently experiencing multiple forms of oppression and marginalisation (Crenshaw, 1989). While health research has historically considered social determinants individually, intersectionality frameworks are now increasingly used to study how multiple forms of marginalisation interact to produce health outcomes (Bowleg, 2012).

Clinical severity was expected to be the factor most strongly associated with admission (Unick *et al*., 2011; Dazzi *et al*., 2015). We assessed the relationship between social determinants and admission before and after adjusting for severity as measured by diagnosis and triage acuity score. Interrelationships were studied within an individual's health context (e.g. comorbidities), and interpreted within the larger socioeconomic and political contexts that produce social determinants of health (Solar and Irwin, 2010).

### Study design and data sources

This population-based cross-sectional study used linked Ontario, Canada health administrative data housed at ICES. ICES is an independent, non-profit research institute whose legal status under Ontario's health information privacy laws allows it to collect and analyse health care and demographic data, without consent, for health system evaluation and improvement. Ontario (population ~14.5 million), has a single-payer healthcare system in which physician and hospital services are government-funded. At ICES, data are available for all provincial residents.

We used the ICES Mothers and Newborns database (linked maternal–infant records for in-hospital births, comprising >98% of Ontario deliveries (Fitzpatrick *et al*., 2021)) to identify postpartum individuals, and the National Ambulatory Care Reporting System to identify the nature and clinical severity of ED visits. We used the Registered Persons Database (demographic data: age, sex, region of residence, neighbourhood income from postal code and Census files), the Immigration, Refugees, and Citizenship Canada Permanent Residents database (immigrations to Canada since 1985 (Chiu *et al*., 2016a)), the Surname-based Ethnicity Group database (Chinese and South Asian ethnicity (Shah *et al*., 2010)) and the Ontario Marginalization Index (On-Marg) (neighbourhood-level ethnic diversity) (Matheson *et al*., 2018) to capture the key social determinants of health under study. For additional variables, we used the Ontario Health Insurance Plan database (physician billings), the Ontario Mental Health Reporting System (psychiatric hospitalisations), the Canadian Institutes of Health Information Discharge Abstract Database (non-psychiatric hospitalisations) (Juurlink *et al*., 2006) and the Institution Information System (hospital-level variables). These datasets are complete and reliable for sociodemographic information and for primary diagnoses in acute care and ambulatory settings (Goel *et al*., 1996). These datasets were linked using unique encoded identifiers and analysed at ICES.

### Participants

We considered all Ontario individuals (female as per their health card) who delivered a live or stillborn infant between 1 April 2008 and 31 March 2016, and had a psychiatric ED visit up to 365 days after childbirth (maximum 31 March 2017). A psychiatric ED visit was defined as an ED visit with a primary psychiatric diagnosis (International Classification of Diseases and Related Health Problems-10, ICD-10 codes F06-F99) or when there was no primary psychiatric diagnosis, but there was evidence of deliberate self-injury coded in any diagnostic field (ICD-10 X60-X84; Y10-Y19; Y28) (MHASEF, 2018). The first psychiatric ED visit following delivery was identified as the index ED visit. For those with multiple deliveries in the study period, one delivery was chosen at random. We excluded individuals who died during the index visit or who left without being seen.

### Study variables

The primary outcome was any hospital admission at the time of the index psychiatric ED visit, as recorded on the ED disposition record. The six social determinants of health available in the ICES datasets were: age, neighbourhood income, immigration category, rurality of residence, Chinese, South Asian or other ethnicity, and neighbourhood diversity (Bonnefoy *et al*., 2007; Huang *et al*., 2007; Barker *et al*., 2016; WHO, 2020). Age was classified into four groups (⩽19, 20–29, 30–39, ⩾40 years) (PHAC, 2009). Neighbourhood income was classified into quintiles using a household size-adjusted measure calculated at the dissemination area (DA) level (400–700 people) (Statistics Canada, 2016, 2019). Immigration category was divided into refugee, non-refugee immigrant and Canadian-born/long-term resident categories. The Statistics Canada definition of rural (communities with populations <10 000) was used to classify individuals as rural or urban residents (du Plessis *et al*., 2001). Self-identified race/ethnicity is not available for most individuals in ICES databases. However, we were able to capture the aspects of ethnicity in two ways. First, Chinese and South Asian ethnicities were captured using validated surname-based algorithms (sensitivity 90.2%/specificity 99.7% for identifying individuals of Chinese ethnicity, sensitivity 50.4%/specificity 99.7% for identifying individuals of South Asian ethnicity, when validated against self-reported ethnicity in the Canadian Community Health Survey (Shah *et al*., 2010)). Although these are just two of many ethnicities represented in Ontario, they are among the most common, with ~6.4% of the population identifying as Chinese and ~8.9% identifying as South Asian (Statistics Canada, 2016). Second, neighbourhood-level ethnic diversity was classified into five quintiles as per the ON-Marg ‘ethnic concentration’ dimension. This DA-level dimension captures the proportion of the population who recently immigrated and/or who self-identify as a visible minority (Matheson *et al*., 2018).

There were two main clinical severity variables: (1) primary psychiatric diagnosis in the ED, and (2) acuity upon presentation to the ED. Primary psychiatric diagnosis was categorised based on the fifth edition of the Diagnostic and Statistical Manual of Mental Disorders (DSM-5) (APA, 2013) as follows: (1) anxiety/obsessive-compulsive/trauma and stressor-related disorders, (2) depressive disorders, (3) bipolar and related disorders, (4) schizophrenia-spectrum/other psychotic disorders, (5) substance-related and addictive disorders or (6) other/non-classified disorders, including deliberate self-harm with a primary non-psychiatric diagnosis (e.g. laceration) (Bethell and Rhodes, 2009). Acuity was measured with the Canadian Triage Assessment Score (CTAS), used in Canadian EDs to triage how urgently a patient needs to be seen and the most appropriate ED treatment area/monitoring level (Beveridge *et al*., 1998). CTAS 1 is ‘resuscitation’, 2 is ‘emergent’ (e.g. acute psychosis/extreme agitation), 3 is ‘urgent’ (e.g. suicidal patients), 4 is ‘less urgent’ (e.g. depression), while 5 is ‘non-urgent’. Herein, CTAS 1–2 was categorised as ‘high acuity’, 3 as ‘moderate acuity’ and 4–5 as ‘low acuity’. For those without primary substance use disorders, we identified comorbid substance use (substance or alcohol-related diagnoses in a non-primary diagnostic field) for inclusion in additional analyses.

Additional variables potentially associated with the likelihood of admission from the ED were: (1) obstetrical (parity; severe maternal morbidity (Ray *et al*., 2018); preterm birth <37 weeks (WHO, 2018)); (2) infant (multi-gestation, stillbirth, neonatal intensive care unit admission; apprehension at birth; death prior to the mother's index visit); (3) maternal medical comorbidity (Charlson score calculated from hospitalisation data) (Quan *et al*., 2005); diagnoses of asthma, diabetes or hypertension using validated definitions (Hux *et al*., 2002; Gershon *et al*., 2009; Quan *et al*., 2009); (4) maternal pre-ED service use (family physician visits (mental health/non-mental health), psychiatrist visits, psychiatric ED visits; psychiatric admissions in the 2 years before delivery); (5) family physician and psychiatrist visits between delivery and the index ED visit (Steele *et al*., 2004); as well as (6) index ED factors, specifically days from birth to the ED visit, visit timing (daytime *v*. evening/overnight), weekend *v*. weekday visit, deliberate self-harm in any diagnostic field (regardless of primary diagnosis) and hospital type (academic/paediatric/mental health *v*. community/small <100 beds).

### Analysis

The sample was described in relation to each social determinant of health separately. For each determinant, we constructed unadjusted and clinical-severity-adjusted modified Poisson regression models estimating relative risk (RR) of admission and 95% confidence intervals (CI) for each level of the variable, using the least marginalised group as the referent (Zou, 2004). We then constructed a fully-adjusted model incorporating all social determinants of health, clinical severity and all other variables. All models used generalised estimating equations to account for hospital-level clustering. Prior to model building, collinearity was assessed using Variance Inflation Factors. As comorbid substance use could also influence the likelihood of admission (Unick *et al*., 2011), an additional analysis among those without primary substance use disorders also adjusted for comorbid substance use as a severity variable.

We used additive interaction to investigate the effect of intersecting social determinants of health on the risk of hospital admission. Additive interaction facilitates an understanding of how the joint effect of two social determinants of health variables differs compared to the sum of their individual effects, and is more relevant to intersectionality and public health than multiplicative interaction (Bauer, 2014). Additive interactions were expressed using the relative excess risk due to interaction (RERI), which represents the effect that is due to interaction (RERI = RR_11_–RR_10_–RR_01_ + 1); RERI > 0 indicates a synergistic effect (i.e. positive interaction, where the observed effect is greater than the sum of the individual effects); RERI < 0 indicates antagonistic effect (i.e. negative interaction); and RERI = 0 indicates no interaction (VanderWeele and Knol, 2014). To ensure sufficient sample size in each category and for ease of interpretation, variables were collapsed into two-level variables. For each two-way variable combination, modified Poisson models with both variables and their multiplicative interaction term were constructed, unadjusted and adjusted for all covariates. RERIs were calculated from the output of the models using SAS code from Zou (2008).

Data use was authorised under section 45 of Ontario's Personal Health Information Protection Act (exempt from Research Ethics Board review). Cells with <6 individuals were suppressed to prevent the identification of individuals. All analyses were done using SAS version 9.4 (SAS Institute, Cary, NC, USA).

## Results

There were 10 859 eligible unique postpartum individuals with psychiatric ED visits (online Supplementary Fig. S1). After excluding 157 (*n* = 1.4%) who were missing data on rurality, income and/or a clinical severity variable, *n* = 10 702 were included. The mean age was 27.4 years (standard deviation 6.3), most individuals were urban-dwelling (82.1%), and the lowest income quintile was disproportionately represented (34.5%). A minority were immigrants (13.9%) and of Chinese (1.7%) or South Asian (2.2% ethnicity). The cohort was relatively balanced across diversity quintiles. Anxiety and related disorders were the most common diagnoses (42.0%), followed by depressive disorders (30.5%). About 27.3% presented with high-acuity, 51.6% with moderate acuity and 21.1% with low-acuity (Table 1).

**Table 1.** Characteristics of 10 702 postpartum with psychiatric ED visits

Variable	*N*(%)
Social determinants of health	
Age in years, 40 or older	333 (3.1)
30–39 years	3638 (34.0)
20–29 year	5566 (52.0)
19 years or under	1165 (10.9)
Income quintile, Q5 (highest)	1229 (11.5)
Q4	1626 (15.2)
Q3	1860 (17.4)
Q2	2296 (21.4)
Q1 (lowest)	3691 (34.5)
Residence, urban	8785 (82.1)
Rural	1916 (17.9)
Immigrant status, Canadian-born/long-term resident	9213 (86.1)
Immigrant (non-refugee)	1176 (11.0)
Refugee	313 (2.9)
Ethnicity, not Chinese or South Asian	10 284 (96.1)
Chinese	183 (1.7)
South Asian	235 (2.2)
Ethnic diversity quintile, Q1 (least diverse)	2215 (20.7)
Q2	2098 (19.6)
Q3	1874 (17.5)
Q4	1801 (16.8)
Q5 (most diverse)	2342 (21.9)
Missing	372 (21.9)
Severity at index ED visit	
Diagnosis, anxiety	4495 (42.0)
Depressive disorders	3269 (30.6)
Bipolar	257 (2.4)
Psychotic	441 (4.1)
Substance use	1242 (11.6)
Other	998 (9.3)
CTAS acuity, low	2258 (21.1)
Moderate	5525 (51.6)
High	2919 (27.3)
Obstetrical, infant and medical^a^	
Primiparous	5000 (46.7)
Multi-gestation	200 (1.9)
Severe maternal morbidity, 1 or more	210 (2.0)
Preterm birth^b^	1229 (11.5)
Stillbirth	165 (1.5)
NICU admission	2149 (20.1)
Infant apprehension	393 (3.7)
Infant death pre-ED	144 (1.4)
Charlson score, 1 or more	979 (9.2)
Asthma	1416 (13.2)
Diabetes	298 (2.8)
Hypertension	300 (2.8)
Health service use pre-delivery^c^	
Family physician non-MH visits, 1 or more	10 215 (95.4)
Family physician MH visits, 1 or more	5283 (49.4)
Psychiatrist visits, 1 or more	2132 (19.9)
MH ED visits, 1 or more	1999 (18.7)
MH admissions, 1 or more	831 (7.8)
Health service use delivery to ED visit^d^	
Family physician non-MH visits, 1 or more	6900 (64.5)
Family physician MH visits, 1 or more	4019 (37.6)
Psychiatrist visits, 1 or more	1157 (10.8)
Index ED visit	
Days from delivery; median (IQR)	153 (64-253)
Evening/overnight visit	6292 (58.8)
Self-harm in any diagnostic field	752 (7.0)
Weekend visit	3000 (28.0)
Community/small hospital	2301 (21.5)

MH, mental health; NICU, neonatal intensive care unit.

a Obstetrical and infant variables are related to the most recent obstetrical delivery.

b Missing = 17.

c In 2 years prior to most recent obstetrical delivery.

d Between most recent obstetrical delivery and index ED visit.

Clinical severity varied by social determinants of health (Table 2). For example, substance use disorders were more common in young individuals and those living in low-income, rural and low-diversity areas, and less common in immigrants/refugees and those of Chinese or South Asian ethnicity. The distribution of the social determinants of health also varied in relation to one another (online Supplementary Table S1). For example, young individuals tended to disproportionately live in lower-income and rural neighbourhoods, and immigrant individuals, those with Chinese and South Asian surnames and those living in more ethnically diverse areas tended to disproportionately live in urban areas. Other characteristics also varied by social determinant (online Supplementary Tables S2a/b). For example, young postpartum individuals, those living in rural and low-income areas, and immigrant and Chinese individuals were all less likely to have been seen by a family physician for mental health care between childbirth and the ED visit than their older, urban, higher-income and Canadian-born/long-term resident, and non-Chinese/South Asian counterparts.

**Table 2.** Clinical severity in relation to social determinants of health, among postpartum individuals with a psychiatric ED visit (*n* = 10 702)

		Age in years				Neighbourhood income quintile					Region of residence	
		40 or older (*n* = 333)	30–39 (*n* = 3638)	20–29 (*n* = 5566)	19 or less (*n* = 1165)	Q5, highest (*n* = 1229)	Q4 (*n* = 1626)	Q3 (*n* = 1860)	Q2 (*n* = 2296)	Q1, lowest (*n* = 3691)	Urban (*n* = 8786)	Rural (*n* = 1916)
Primary diagnosis^a^	Anxiety	140 (42.0)	1556 (42.8)	2334 (41.9)	465 (39.1)	520 (42.3)	721 (44.3)	825 (44.4)	948 (41.3)	1481 (40.1)	3651 (41.6)	844 (44.0)
	Depressive	101 (30.3)	1207 (33.2)	1622 (29.1)	339 (29.1)	426 (34.7)	543 (33.4)	583 (31.3)	712 (31.0)	1005 (27.2)	2713 (30.9)	556 (29.0)
	Bipolar	12 (3.6)	106 (2.9)	127 (2.3)	12 (1.0)	28 (2.3)	40 (2.5)	35 (1.9)	69 (3.0)	85 (2.3)	240 (2.7)	17 (0.9)
	Psychotic	30 (9.0)	201 (5.5)	188 (3.4)	22 (1.9)	49 (4.0)	48 (3.0)	74 (4.0)	100 (4.4)	170 (4.6)	417 (4.8)	24 (1.2)
	Substance use	24 (7.2)	279 (7.7)	768 (13.8)	171 (14.7)	104 (8.5)	133 (8.2)	183 (9.8)	277 (12.1)	545 (14.8)	941 (10.7)	301 (15.7)
	Other	26 (7.8)	289 (7.9)	527 (9.5)	156 (13.4)	102 (8.3)	141 (8.67)	160 (8.6)	190 (8.3)	405 (11.0)	824 (9.4)	174 (9.1)
Acuity	Low	52 (15.6)	641 (17.6)	1279 (23.0)	286 (24.6)	272 (22.1)	318 (19.6)	364 (19.6)	449 (19.6)	855 (23.2)	1464 (16.7)	794 (41.4)
	Moderate	187 (56.2)	1909 (52.5)	2878 (51.7)	551 (47.3)	625 (50.8)	862 (53.0)	964 (51.8)	1197 (52.1)	1877 (50.8)	4664 (53.1)	861 (44.9)
	High	94 (28.2)	1088 (29.9)	1409 (25.3)	328 (28.2)	332 (27.0)	446 (27.4)	532 (28.6)	650 (28.3)	959 (26.0)	2658 (30.2)	261 (13.6)
		**Immigrant status**			**Ethnicity**			**Ethnic diversity quintile (Q)**^b^				
		Long-term resident (*n* = 9213)	Non-refugee (*n* = 1176)	Refugee (*n* = 313)	Neither (*n* = 10 284)	Chinese (*n* = 183)	South Asian (*n* = 235)	Q1, most (*n* = 2215)	Q2 (*n* = 2098)	Q3 (*n* = 1874)	Q4 (*n* = 1801)	Q5, least (*n* = 2342)
Primary diagnosis^a^	Anxiety	3867 (42.0)	482 (41.0)	146 (46.6)	4334 (42.1)	64 (35.0)	97 (41.3)	1014 (45.8)	929 (44.3)	785 (41.9)	728 (40.4)	927 (39.6)
	Depressive	2796 (30.4)	399 (33.9)	74 (23.6)	3122 (30.4)	68 (37.2)	79 (33.6)	676 (30.5)	639 (30.5)	586 (31.3)	577 (32.0)	728 (31.1)
	Bipolar	219 (2.4)	27 (2.3)	11 (3.51)	246 (2.39)	NR	NR	174 (7.9)	173 (8.2)	164 (8.8)	171 (9.5)	253 (10.8)
	Psychotic	318 (3.4)	91 (7.7)	32 (10.2)	404 (3.93)	18 (9.84)	19 (8.09)	NR	42 (2.0)	57 (3.0)	49 (2.7)	74 (2.9)
	Substance use	1201 (13.0)	31 (2.6)	10 (3.2)	1228 (11.9)	NR	NR	272 (12.3)	254 (12.1)	213 (11.4)	186 (10.3)	188 (8.0)
	Other	812 (8.8)	146 (12.4)	40 (12.8)	950 (9.24)	22 (12.0)	26 (11.1)	NR	61 (2.9)	69 (3.7)	90 (5.0)	172 (7.3)
Acuity	Low	2074 (22.5)	141 (12.0)	43 (13.7)	2198 (21.4)	34 (18.6)	26 (11.1)	636 (28.7)	544 (25.9)	331 (17.7)	294 (16.3)	255 (10.9)
	Moderate	4752 (51.6)	622 (52.9)	151 (48.2)	5312 (51.6)	76 (41.5)	137 (58.3)	1111 (50.2)	1048 (50.0)	1012 (54.0)	948 (52.6)	1272 (54.3)
	High	2387 (25.9)	413 (35.1)	119 (38.0)	2774 (27.0)	73 (39.9)	72 (30.6)	468 (21.1)	506 (24.1)	531 (28.3)	559 (31.0)	815 (34.8)

a Diagnostic categories: anxiety, obsessive-compulsive and trauma- and stressor-related disorders; depressive disorders; bipolar and related disorders; schizophrenia spectrum and other psychotic disorder; substance-related and addictive disorders; other/non-classified disorders.

b *N* = 372 missing; NR = not reportable (all cells with <6 individuals or from which such cells could be calculated are suppressed as per ICES guidelines).

**Fig. 2.**
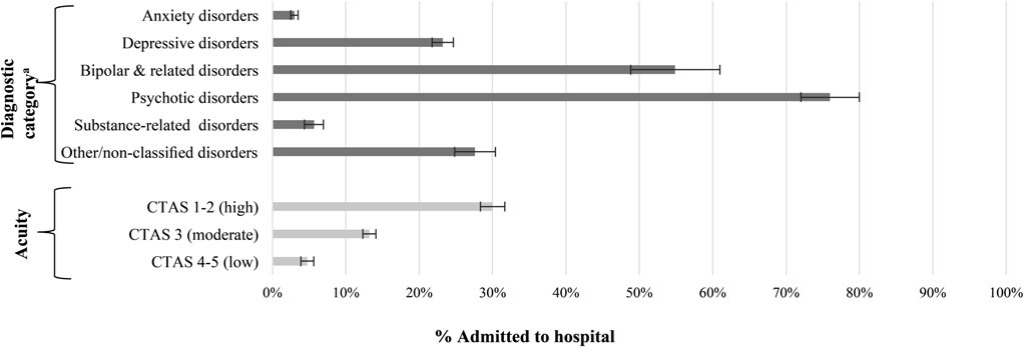
Admission to hospital at the time of index ED visit by diagnostic category and acuity at triage, presented as % and 95% confidence interval (bars). ^a^Diagnostic categories: Anxiety, obsessive-compulsive, and trauma- and stressor-related disorders; Depressive disorders; Bipolar and related disorders; Schizophrenia spectrum and other psychotic disorder; Substance-related and addictive disorders; Other/non-classified disorders.

Overall, 16.0% of the cohort (*n* = 1715) was admitted to hospital, from 3.0% of those with anxiety, obsessive-compulsive and trauma-related disorder presentations to 76.0% of those with schizophrenia spectrum and other psychotic disorder presentations (Fig. 2). About 4.7% of those with low-acuity were admitted, 13.2% with moderate-acuity and 30.0% with high-acuity.

All social determinants of health were associated with the likelihood of admission in unadjusted models (Table 3). Being younger *v*. older, rural *v*. urban-dwelling and living in lower-income *v*. higher-income areas were each associated with a lower likelihood of admission. Being a non-refugee immigrant *v*. Canadian-born/a long-term resident, Chinese *v*. non-Chinese or South Asian ethnicity, and living in a more *v*. less ethnically diverse neighbourhood were each associated with greater likelihood. Effects observed were attenuated in most cases after adjusting for clinical severity, and only Chinese ethnicity was significantly associated with a higher risk of admission in fully-adjusted models (aRR 1.49, 95% CI 1.24–1.80).

**Table 3.** Risk of admission within each social determinant of health; presented as crude risk, adjusted for clinical severity (diagnosis and acuity at triage) and fully-adjusted for all covariates

Social determinant of health	Admitted *N* (%)	Crude RR (95% CI)	Severity-adjusted RR (95% CI)	Fully-adjusted^a^ RR (95% CI)
Age in years				
40 or older (*n* = 333)	79 (23.7)	1.00 (referent)	1.00 (referent)	1.00 (referent)
30–39 years (*n* = 3638)	679 (18.7)	**0.**7**9** (**0.66–0.95)**	0.89 (0.78**–**1.02)	0.91 (0.79**–**1.05)
20–29 year (*n* = 5566)	802 (14.4)	**0.64** (**0.52–0.78)**	**0.84** (**0.72–0.98)**	0.89 (0.76**–**1.05)
19 years or under (*n* = 1165)	155 (13.3)	**0.60** (**0.45–0.82)**	0.83 (0.67**–**1.04)	0.90 (0.72**–**1.12)
Income quintile				
Q5 (highest) (*n* = 1229)	221 (18.0)	1.00 (referent)	1.00 (referent)	1.00 (referent)
Q4 (*n* = 1626)	264 (16.2)	0.88 (0.73**–**1.06)	0.95 (0.82**–**1.10)	0.96 (0.82**–**1.10)
Q3 (*n* = 1860)	296 (15.9)	0.86 (0.71**–**1.06)	0.91 (0.79**–**1.06)	0.93 (0.80**–**1.08)
Q2 (*n* = 2296)	382 (16.6)	0.89 (0.72**–**1.09)	0.91 (0.78**–**1.06)	0.92 (0.79**–**1.08)
Q1 (lowest) (*n* = 3691)	552 (15.0)	**0.81** (**0.66–0.98)**	**0.85** (**0.74–0.98)**	0.90 (0.77**–**1.05)
Immigrant category				
Canadian-born/long-term resident (*n* = 9213)	1380 (15.0)	1.00 (referent)	1.00 (referent)	1.00 (referent)
Immigrant (non-refugee) (*n* = 1176)	269 (22.9)	1**.29** (**1.06–1.56)**	1.05 (0.92**–**1.20)	1.11 (0.98**–**1.26)
Refugee (*n* = 313)	66 (21.1)	1.19 (0.86**–**1.66)	0.95 (0.78**–**1.16)	1.05 (0.87**–**1.26)
Residence				
Urban (*n* = 8785)	1523 (17.3)	1.00 (referent)	1.00 (referent)	1.00 (referent)
Rural (*n* = 1916)	192 (10.0)	**0.75** (**0.62–0.91)**	1.04 (0.88**–**1.22)	1.06 (0.90**–**1.26)
Ethnicity				
Not Chinese or South Asian (*n* = 10 284)	1601 (15.6)	1.00 (referent)	1.00 (referent)	1.00 (referent)
Chinese (*n* = 183)	63 (34.4)	**1.88** (**1.42–2.49)**	**1.43** (**1.56–1.77)**	**1.49** (**1.24–1.80)**
South Asian (*n* = 235)	51 (21.7)	1.15 (0.84**–**1.57)	1.03 (0.80**–**1.32)	1.05 (0.82**–**1.35)
Ethnic diversity quintile				
Q1 (least diverse) (*n* = 2215)	272 (12.3)	1.00 (referent)	1.00 (referent)	1.00 (referent)
Q2 (*n* = 2098)	316 (15.1)	**1.18** (**1.01–1.37)**	1.07 (0.94**–**1.21)	1.07 (0.94**–**1.22)
Q3 (*n* = 1874)	329 (17.6)	**1.29** (**1.12–1.50)**	1.08 (0.96**–**1.22)	1.10 (0.96**–**1.25)
Q4 (*n* = 1801)	300 (16.7)	1.16 (0.96**–**1.39)	0.93 (0.81**–**1.07)	0.92 (0.80**–**1.05)
Q5 (most diverse) (*n* = 2342)	473 (20.2)	**1.24** (**1.01–1.53)**	0.91 (0.79**–**1.06)	0.91 (0.79**–**1.03)
Missing (*n* = 372)	25 (6.72)	**0.55** (**0.32–0.95)**	0.82 (0.53**–**1.24)	0.90 (0.79**–**1.03)

a Adjusted for diagnosis, acuity, SDOH and all other variables. Bolded font denotes statistical significance. See online Supplementary Table S3 for the full adjusted model.

Additional variables associated with an increased risk of admission in the fully-adjusted model were prior mental health outpatient care (before and after delivery), psychiatric admission in the 2 years prior to delivery, and the timing of the ED visit being in the evening/overnight (*v*. in the daytime); conversely, multigestation (*v*. singleton) birth and pre-delivery psychiatric ED visits were associated with a lower risk of admission (online Supplementary Table S3).

No measures of additive interaction between social determinants of health were statistically significant in the 15 combinations explored (Table 4).

**Table 4.** Additive interactions examining the impact of combinations of social determinants of health on the risk of admission at the time of index ED presentation, expressed as RERI (95% CI)

Interaction model	Unadjusted additive RERI (95% CI)	Adjusted × additive RERI (95% CI)
Young age × rural	0.07 (−0.25 to 0.40)	0.004 (−0.55 to 0.85)
Young age × low income	−0.23 (−0.54 to 0.09)	−0.12 (−0.50 to 0.21)
Young age × immigrant	−0.39 (−1.10 to 0.31)	−0.28 (−0.82 to 0.92)
Young age × diverse area	0.09 (−0.24 to 0.38)	−0.01 (−0.44 to 0.36)
Young age × Chinese/South Asian	Unable to compute	0.06 (−1.12 to 8.19)
Low income × immigrant	0.27 (−0.015 to 0.53)	0.13 (−0.13 to 0.38)
Low income × Chinese/South Asian	−0.17 (−0.73 to 0.36)	−0.16 (−0.65 to 0.31)
Low income × diverse area	Unable to compute	*−*0.02 (−0.24 to 0.18)
Rural × low income	−0.11 (−0.35 to 0.11)	0.09 (−0.24 to 0.42)
Rural × immigrant	−0.33 (−1.14 to 0.48)	−0.38 (−0.98 to 1.31)
Rural × Chinese/South Asian	0.30 (−0.87 to 4.72)	0.68 (−0.85 to 6.69)
Rural × diverse area	0.36 (−2.33 to 0.77)	0.35 (−3.93 to 0.99)
Immigrant × Chinese/South Asian	−0.57 (−1.29 to 0.04)	−0.49 (−1.14 to 0.06)
Immigrant × diverse area	−0.09 (−0.48 to 0.38)	−0.20 (−0.56 to 0.24)
Chinese/South Asian × diverse area	−0.20 (−0.86 to 0.79)	0.04 (−0.63 to 1.25)

Young age = age 19 years and under (*v*. 20 years and over); low income = quintiles 1 and 2 (*v*. quintiles 3–5); Immigrant = immigrant including refugee (*v*. non-immigrant); diverse area = quintiles 4 and 5 (*v*. quintiles 1–3); note missing data not included.

In the additional analysis of individuals without primary substance use disorders (*n* = 9460), 192 (2.0%) had a documented comorbid substance use disorder. Within this cohort, the effects of the social determinants of health were similar to those in the main cohort, and the inclusion of comorbid substance use as a clinical severity variable in addition to primary diagnosis and acuity resulted in a similar attenuation of these effects in both the clinical severity-adjusted and fully-adjusted models (online Supplementary Table S4). Comorbid substance use was independently associated with the likelihood of admission (fully adjusted RR 1.42, 95% CI 1.18–1.71).

## Discussion

In the postpartum period, we found that those who were young, living in rural areas or in low-income neighbourhoods were less likely to be admitted to hospital at the time of a psychiatric ED visit than their older, urban-dwelling and higher-income-neighbourhood counterparts. We also found that immigrants, those of Chinese ethnicity, and those residing in ethnically-diverse areas were more likely to be admitted than their counterparts who were Canadian-born or long-term residents, not Chinese or South Asian, or those living in low ethnic diversity neighbourhoods. These differences were largely explained by variation in clinical severity across social determinants of health. However, individuals of Chinese ethnicity remained more likely to be admitted than non-Chinese counterparts after adjusting for clinical and other measured factors. Interestingly, we did not find evidence of intersecting relationships between social determinants and the likelihood of admission, suggesting that the impact of one determinant does not necessarily depend on a person's level of another determinant (e.g. that the impact of age does not necessarily depend on a person's income level, or *vice versa*).

For most social determinants, the effects in our study attenuated after adjustment for clinical factors. In some non-perinatal populations, younger and lower-income individuals are less frequently admitted to hospital from the ED, even after adjusting for other factors, but there are no comparable data in postpartum populations (Unick *et al*., 2011; Bahji *et al*., 2020). Our finding that those of Chinese ethnicity had an increased likelihood of admission from the ED *after* accounting for clinical severity is consistent with an American study that found an increased adjusted odds of admission among Asian individuals, although that study did not differentiate between various East and South Asian ethnicities (Unick *et al*., 2011). One prior non-perinatal study examined intersecting social determinants by stratifying predictive models of admission stratified by race/ethnicity (Unick *et al*., 2011). Some admission predictors differed by strata (e.g. being employed increased admission likelihood among white and not Asian individuals), although that study did not examine intersections of other determinants. It is possible that our study results differ because there are interactions between other social determinants that we were not able to capture (e.g. race).

The mechanisms that underlie our findings are not necessarily discernable from health administrative data. However, the young, lower-income and rural-dwelling individuals in our study with lower admission risk were disproportionately more likely than their older, higher-income and urban-dwelling counterparts to have low-severity presentations (i.e. lower CTAS scores). They were also less likely to present with the severe mental illnesses such as bipolar disorder and schizophrenia that often require admission. These individuals also had low rates of pre-ED mental health care, raising the question of whether some might never have come to the ED if they had better outpatient access. Conversely, immigrant individuals were more likely to be admitted, which was explained by their disproportionate likelihood of severe clinical presentations to the ED. These individuals also had low rates of pre-ED mental health care, raising the question of whether delays in outpatient care led to more severe presentations that could have been avoided with improved access. In prior research, postpartum individuals who are young, low-income, rurally-dwelling and immigrants may all experience poor access to mental health services, supporting this hypothesis (Hodgkinson *et al*., 2014; Barker *et al*., 2016; Vigod *et al*., 2016; Ta Park *et al*., 2019).

The reason why individuals of Chinese ethnicity were more likely to be admitted, even after adjusting for other measured sociodemographic and clinical factors, is harder to explain. While individuals of Chinese ethnicity represent >6% of the Ontario population, less than 2% of postpartum psychiatric ED visits were among those of Chinese ethnicity (Statistics Canada, 2016); there may be something different in the postpartum ED utilisation patterns of Chinese individuals which also influences admission patterns. While we could account for overall severity, there may have been other differences in clinical presentation contributing to admission likelihood that we were not able to capture. For example, prior non-perinatal Canadian work has found that, among those with psychiatric admissions, individuals of Chinese ethnicity are more likely to exhibit positive symptoms of psychosis (Chiu *et al*., 2016*a*, *b*); triage acuity scores may not have picked up this subtlety in specific symptomatology. There may also be factors related to individual preference for admission and/or factors contributing to differential treatment different in the ED (i.e. implicit, explicit or structural biases) that we were not able to capture herein (McKenzie and Bhui, 2007; Fitzgerald and Hurst, 2017). More research is needed to elucidate this further.

Strengths of this study include the large population-based cohort of postpartum individuals, the multiple social determinants examined (alone and in combination) and the broad range of covariates. There were limitations common to health administrative data, including that income was captured using neighbourhood income quintile which, although measured in a small area, may not reflect individual income, that we did not have data on other important social determinants including gender identity, sexuality, education, employment, marital status or disability, and that we could not measure all aspects of clinical severity. For example, comorbid substance use was based on secondary diagnoses, which may be incomplete in ED data. Due to low numbers of immigrant and Chinese/South Asian individuals and diverse neighbourhoods in rural areas, these variables may not be fully accounted for in the adjusted estimates and interactions for rural residents. A major limitation is the lack of data on race and Indigenous status, and the limited individual-level data on ethnicity. Neither could we measure systemic structural factors such as sociopolitical contexirts that lead to marginalisation and discrimination of specific groups (Solar and Irwin, 2010) or related provider biases. For example, immigrants may be misdiagnosed with psychotic disorders (Adeponle *et al*., 2012), and acuity level at triage that includes subjectivity on the clinician's part may introduce implicit (or explicit) bias within triage acuity scores. Further research is warranted to more fully understand the patterns of care amongst racially and ethnically minoritised groups. We hope that in the future our jurisdiction will collect reliable race- and ethnicity-based data to improve the capacity for equity-oriented health services research.

## Conclusion

This study aimed to understand the associations between social determinants of health and postpartum psychiatric admission, using an intersectionality frame to understand how admission patterns might differ for postpartum individuals marginalised in multiple ways. We found that while the likelihood of being admitted to hospital at the time of an ED visit varied by age, neighbourhood income, urban/rural residence, immigrant category and neighbourhood diversity, these differences were largely explained by differences in diagnosis and acuity at presentation. This suggests that the severity of illness appears to be the overarching driver of the decision to admit in most cases. However, there was a higher likelihood of admission among Chinese individuals independent of other clinical and sociodemographic factors, the mechanism for which warrants further study. In contrast to our original hypothesis, we did not find interactions between social determinants of health related to the admission outcome. However, even when synergistic or antagonistic effects are not at play, individuals experience the sum of the effects of multiple forms of marginalisation. As such, considering social determinants of health collectively is important when considering strategies to improve postpartum mental health care, to ensure that services meet the needs of individuals marginalised in multiple ways.

## Data Availability

The data set from this study is held securely in coded form at ICES. While data sharing agreements prohibit ICES from making the data set publicly available, access may be granted to those who meet pre-specified criteria for confidential access, available at www.ices.on.ca/DAS. The full dataset creation plan is available from the authors upon request, understanding that the programmes may rely upon coding templates or macros that are unique to ICES. The ICES graduate student (LCB) had full access to study data.

## Supplementary material

For supplementary material accompanying this paper visit http://dx.doi.org/ 10.1017/S2045796021000238. click here to view supplementary material
