# Supplementary material for: Hospital admission at the time of a postpartum psychiatric emergency department visit: the influence of the social determinants of health
Source: Epidemiol Psychiatr Sci. 2021 Apr 23;30:e33. doi: 10.1017/S2045796021000238 (PMC8157505; doi:10.1017/S2045796021000238)
Supplement: Supplementary file 1 [file S2045796021000238sup001.docx]

**Figure S1**. Cohort creatio

Exclusions:

Missing data rurality or neighbourhood income quintile

N=109

Missing data on a severity variable

N=48

Study exclusions:

Multiple deliveries in the study period (excluded one delivery at random)

N=567

Died during index ED visit or left without being seen

N=379

**Final cohort**

N=10,702

Eligible unique individuals

N= 10,859

Index MH ED visits for postpartum individuals

N= 11,238

**Table S1.** Social determinants of health and their relation to each other among postpartum individuals with a psychiatric ED visit (n=10,702).

| Variable | Level | Age in years | | | | | Neighbourhood Income Quintile | | | | | Region of Residence | |
| --- | --- | --- | --- | --- | --- | --- | --- | --- | --- | --- | --- | --- | --- |
|  |  | **40 or older**  **(n=333)** | **30-39**  **(n=3,638)** | **20-29 y**  **(n=5,566)** | | **19 or less**  **(n=1,165)** | **Q5**  **(n=1,229)** | **Q4**  **(n=1,626)** | **Q3**  **(n=1,860)** | **Q2**  **(n=2,296)** | **Q1**  **(n=3,691)** | **Urban**  **(n=8,786)** | **Rural**  **(n=1,916)** |
| Age | **40 or older** |  |  |  | |  | 53 (4.31) | 54 (3.32) | 62 (3.33) | 68 (2.96) | 96 (2.60) | 297 (3.4) | 36 (1.9) |
|  | **30-39** |  |  |  | |  | 557 (45.3) | 686 (42.2) | 726 (39.0) | 717 (31.2) | 952 (25.8) | 3162 (36.0) | 476 (24.8) |
|  | **20-29** |  |  |  | |  | 537 (43.7) | 745 (45.8) | 912 (49.0) | 1234 (53.8) | 2138 (57.9) | 4428 (79.6) | 1138 (59.4) |
|  | **19 or under** |  |  |  | |  | 82 (6.67) | 141 (8.67) | 160 (8.60) | 277 (12.1) | 505 (13.7) | 899 (10.2) | 266 (13.9) |
| Income Quintile (Q) | **Q5 (highest)** | 53 (15.9) | 557 (15.3) | 537 (9.65) | | 82 (7.04) |  |  |  |  |  | 955 (10.9) | 274 (14.3) |
|  | **Q4** | 54 (16.2) | 686 (18.9) | 745 (13.4) | | 141 (12.1) |  |  |  |  |  | 1348 (15.3) | 278 (14.5) |
|  | **Q3** | 62 (18.6) | 726 (20.0) | 912 (16.4) | | 160 (13.7) |  |  |  |  |  | 1539 (17.5) | 321 (16.8) |
|  | **Q2** | 68 (20.4) | 717 (19.7) | 1234 (22.2) | | 277 (23.8) |  |  |  |  |  | 1932 (22.0) | 364 (19.0) |
|  | **Q1 (lowest)** | 96 (28.8) | 952 (26.2) | 2138 (38.4) | | 505 (43.4) |  |  |  |  |  | 3012 (34.3) | 679 (35.4) |
| Residence | **Urban** | 297(89.2) | 3162 (86.9) | 4428 (79.6) | | 899 (77.2) | 955 (77.7) | 1348 (82.9) | 1539 (82.7) | 1932 (84.2) | 3012 (81.6) |  |  |
|  | **Rural** | 36 (10.8) | 476 (13.1) | 1138 (20.4) | | 266 (22.8) | 274 (22.3) | 278 (17.1) | 321 (17.3) | 364 (15.8) | 679 (18.4) |  |  |
| Immigrant category | **Canadian-born/ LTR** | 249(74.8) | 2881 (79.2) | 4954 (89.0) | | 1129 (96.9) | 1088 (88.5) | 1402 (86.2) | 1592 (85.6) | 1970 (85.8) | 3161 (85.6) | 7330 (83.4) | 1883 (98.3) |
|  | **Non-refugee** | 62 (18.6) | 631 (17.3) | 464 (8.3) | | 19 (1.6) | 121 (9.8) | 186 (11.4) | 217 (11.7) | 270 (11.8) | 382 (10.4) | NR | NR |
|  | **Refugee** | 22 (6.6) | 126 (3.5) | 148 (2.7) | | 17 (1.5) | 20 (1.6) | 38 (2.3) | 51 (2.7) | 56 (2.4) | 148 (4.0) | NR | NR |
| Ethnicity | **Neither** | 309 (92.8) | 3392 (93.2) | 5424 (97.4) | | 1159 (99.5) | 1180 (96.0) | 1546 (95.1) | 1786 (96.0) | 2191 (95.4) | 3581 (97.0) | 8377 (95.3) | 1907 (99.5) |
|  | **Chinese** | NR | 115 (3.2) | 53 (1.0) | | NR | 32 (2.60) | 40 (2.46) | 20 (1.08) | 46 (2.00) | 45 (1.22) | NR | NR |
|  | **South Asian** | NR | 131 (3.6) | 89 (1.6) | | NR | 17 (1.38) | 40 (2.46) | 54 (2.90) | 59 (2.57) | 65 (1.76) | NR | NR |
| Ethnic diversity Quintile (Q) | **Q1 (least diverse)** | NR | NR | NR | | NR | NR | NR | NR | NR | NR | NR | NR |
|  | **Q2** | 48 (14.4) | 614 (16.9) | 1302 (23.4) | | 276 (23.7) | 314 (25.6) | 353 (21.7) | 364 (19.6) | 517 (22.5) | 550 (14.9) | 1626 (18.5) | 472 (24.6) |
|  | **Q3** | 59 (17.7) | 638 (17.5) | 945 (17.0) | | 232 (19.9) | 307 (25.0) | 307 (18.9) | 347 (18.7) | 390 (17.0) | 523 (14.2) | 1772 (20.2) | 472 (24.6) |
|  | **Q4** | 66 (19.8) | 729 (20.0) | 836 (15.0) | | 170 (14.6) | 254 (20.7) | 312 (19.2) | 333 (17.9) | 357 (15.6) | 545 (14.8) | 1785 (20.3) | 16 (0.8) |
|  | **Q5 (most diverse)** | 105 (31.5) | 1003 (27.6) | 1096 (19.7) | | 138 (11.9) | 90 (7.3) | 281 (17.3) | 371 (20.0) | 486 (21.2) | 1114 (30.2) | NR | NR |
|  |  | **Immigrant status** | | | | **Ethnicity** | | | **Ethnic diversity quintile (Q)^a^** | | | | |
|  |  | **Canadian-born/Long-term resident (n=9,213)** | **Non-refugee**  **(n=1,176)** | **Refugee**  **(n=313)** | | **Neither**  **(n=10,284)** | **Chinese**  **(n=183)** | **South Asian**  **(n=235)** | **Q1**  **(n= 2215)** | **Q2**  **(n=2098)** | **Q3**  **(n=1874)** | **Q4**  **(n=1801)** | **Q5  (n=2342)** |
| Age | **40 or older** | 249 (2.70) | 62 (5.27) | 22 (7.03) | | 309 (3.00) | NR | NR | NR | 48 (2.3) | 59 (3.2) | 66 (3.7) | 105 (4.5) |
|  | **30-39** | 2881 (31.3) | 631 (53.7) | 126 (40.3) | | 3392 (33.0) | 115 (62.8) | 131 (55.7) | NR | 614 (29.3) | 638 (34.0) | 729 (40.5) | 1003 (42.8) |
|  | **20-29** | 4954 (53.8) | 464 (39.5) | 148 (47.3) | | 5424 (52.7) | 53 (29.0) | 89 (37.9) | NR | 1153 (55.0) | 945 (50.4) | 836 (46.4) | 1096 (46.8) |
|  | **19 or under** | 1129 (12.2) | 19 (1.62) | 17 (5.43) | | 1159 (11.3) | NR | NR | NR | 283 (13.5) | 232 (12.4) | 170 (9.4) | 138 (5.9) |
| Income quintile (Q) | **Q5 (highest)** | 1088 (11.8) | 121 (10.3) | 20 (6.39) | | 1180 (11.5) | 32 (17.5) | 17 (7.23) | NR | 314 (15.0) | 307 (16.4) | 254 (14.1) | 90 (3.8) |
|  | **Q4** | 1402 (15.2) | 186 (15.8) | 38 (12.1) | | 1546 (15.0) | 40 (21.9) | 40 (17.0) | NR | 353 (16.8) | 307 (16.4) | 312 (17.3) | 281 (12.0) |
|  | **Q3** | 1592 (17.3) | 217 (18.4) | 51 (16.3) | | 1786 (17.4) | 20 (10.9) | 54 (23.0) | NR | 364 (17.4) | 347 (18.5) | 333 (18.5) | 371 (15.8) |
|  | **Q2** | 1970 (21.4) | 270 (23.0) | 56 (17.9) | | 2191 (21.3) | 46 (25.1) | 59 (25.1) | NR | 517 (24.6) | 390 (20.8) | 357 (19.8) | 486 (20.8) |
|  | **Q1 (lowest)** | 3161 (34.3) | 382 (32.5) | 148 (47.3) | | 3581 (34.8) | 45 (24.6) | 65 (27.7) | NR | 550 (26.2) | 523 (27.9) | 545 (30.3) | 1114 (47.6) |
| Residence | **Urban** | 7330 (79.6) | NR | NR | | 8377 (81.5) | NR | NR | NR | 1626 (77.5) | 1772 (94.6) | 1785 (99.1) | NR |
|  | **Rural** | 1883 (20.4) | NR | NR | | 1907 (18.5) | NR | NR | NR | 472 (22.5) | 102 (5.4) | 16 (0.9) | NR |
| Immigrant status | **Canadian-born/ LTR** |  |  |  | | 9064 (88.1) | 72 (39.3) | 77 (32.8) | NR | NR | 1759 (93.9) | 1497 (83.1) | 1381 (59.0) |
|  | **Non-refugee** |  |  | |  | 941 (9.15) | 97 (53.0) | 138 (58.7) | NR | NR | 97 (5.2) | 238 (13.2) | 746 (31.9) |
|  | **Refugee** |  |  | |  | 279 (2.71) | 14 (7.65) | 20 (8.51) | NR | NR | 18 (1.0) | 66 (3.7) | 215 (9.2) |
| Ethnicity | **Neither** | 9064 (98.4) | 941 (80.0) | | 279 (89.1) |  |  |  | NR | 2081 (99.2) | 1845 (98.4) | 1741 (96.7) | 2049 (87.5) |
|  | **Chinese** | 72 (0. 8) | 97 (8.2) | | 14 (4.5) |  |  |  | NR | NR | 15 (0.8) | 27 (1.5) | 123 (5.2) |
|  | **South Asian** | 77 (0.8) | 138 (11.0) | | 20 (6.4) |  |  |  | NR | NR | 14 (0.4) | 33 (1.8) | 170 (7.3) |
| Ethnic diversity quintile (Q) | **Q1 (least diverse)** | NR | NR | | NR | NR | NR | NR |  |  |  |  |  |
|  | **Q2** | NR | NR | | NR | 2081 (20.2) | NR | NR |  |  |  |  |  |
|  | **Q3** | 1759 (19.1) | 97 (8.2) | | 18 (5.8) | 1845 (17.9) | 15 (8.2) | 14 (6.0) |  |  |  |  |  |
|  | **Q4** | 1497 (16.2) | 238 (20.2) | | 66 (21.1) | 1741 (16.9) | 27 (14.8) | 33 (14.0) |  |  |  |  |  |
|  | **Q5 (most diverse)** | 1381 (15.0) | 746 (63.4) | | 215 (68.7) | 2049 (19.9) | 123 (67.2) | 170 (72.3) |  |  |  |  |  |

NR=Not reportable (all cells with <6 individuals or from which such cells could be calculated are suppressed as per ICES guidelines); LTR=Long-term resident;  ^a^N=372 Missing

Table S2a. Additional baseline characteristics, presented by social determinant of health for age, neighbourhood income quintile, and residence.

|  | **Age** | | | | **Neighbourhood Income Quintile (Q)** | | | | | **Residence** | |
| --- | --- | --- | --- | --- | --- | --- | --- | --- | --- | --- | --- |
|  | **40 or older**  **(n=333)** | **30-39**  **(n=3,638)** | **20-29**  **(n=5,566)** | **19 or less**  **(n=1,165)** | **Q5**  **(Highest)**  **(n=1,229)** | **Q4**  **(n=1,626)** | **Q3**  **(n=1,860)** | **Q2**  **(n=2,296)** | **Q1**  **(Lowest) (n=3,691)** | **Urban**  **(n=8,786)** | **Rural**  **(n=1,916)** |
| **Obsetrical^a^, Infant, and Medical** |  |  |  |  |  |  |  |  |  |  |  |
| Primiparous | 97 (29.1) | 1254 (34.5) | 2637 (47.4) | 1012 (86.9) | 622 (50.6) | 816 (50.2) | 885 (47.6) | 1088 (47.4) | 1589 (43.0) | 4189 (47.7) | 811 (42.3) |
| SMM ≥1 | 6 (1.8) | 68 (1.9) | 114 (2.0) | 22 (1.9) | 23 (1.9) | 34 (2.1) | 33 (1.8) | 45 (2.0) | 75 (2.03) | 171 (1.95) | 45 (2.4) |
| Preterm birth^b^ | NR | NR | NR | NR | NR | NR | NR | NR | NR | NR | NR |
| Multi-gestation | 12 (3.6) | 94 (2.6) | 83 (1.5) | 11 (0.9) | 28 (2.3) | 31 (1.9) | 40 (2.2) | 41 (1.8) | 60 (1.6) | 165 (1.88) | 29 (1.5) |
| Stillbirth | 14 (4.2) | 57 (1.6) | 76 (1.4) | 18 (1.6) | 15 (1.2) | 13 (0.8) | 34 (1.8) | 38 (1.7) | 65 (1.8) | 120 (1.37) | 45 (2.4) |
| NICU admission | 72 (23.4) | 682 (19.5) | 1050 (19.4) | 226 (19.9) | 197 (16.6) | 256 (16.2) | 335 (18.8) | 436 (19.6) | 806 (22.6) | 1756 (20.7) | 274 (14.9) |
| Infant apprehension | 12 (3.9) | 94 (2.7) | 206 (3.8) | 62 (5.5) | 17 (1.4) | 27 (1.7) | 56 (3.1) | 80 (3.6) | 194 (5.4) | 329 (3.87) | 45 (2.4) |
| Infant death pre-ED | 6 (2.0) | 45 (1.3) | 66 (1.2) | 13 (1.1) | 15 (1.3) | 10 (0.6) | 21 (1.9) | 35 (1.6) | 49 (1.37) | 100 (1.18) | 30 (1.6) |
| Charlson score > 1 | 43 (12.9) | 358 (9.8) | 499 (9.0) | 79 (6.8) | 90 (7.3) | 124 (7.6) | 144 (7.7) | 220 (9.6) | 401 (10.9) | 802 (82.1) | 177 (9.2) |
| Asthma | 31 (9.3) | 328 (9.0) | 798 (14.3) | 31 (9.3) | 125 (10.2) | 199 (12.2) | 223 (12.0) | 306 (13.3) | 563 (15.2) | 1205 (13.7) | 211 (11.0) |
| Diabetes | 25 (7.5) | 146 (4.0) | 117 (2.1) | 25 (7.5) | 35 (2.9) | 44 (2.7) | 48 (2.6) | 57 (2.5) | 114 (3.1) | 258 (2.94) | 40 (2.1) |
| Hypertension | 30 (9.0) | 203 (5.6) | 67 (1.2) | 30 (9.0) | 46 (3.7) | 54 (3.3) | 54 (2.9) | 58 (2.5) | 88 (2.4) | 265 (3.02) | 35 (11.7) |
| **Prior Service Use** |  |  |  |  |  |  |  |  |  |  |  |
| ***Pre-delivery^c^*** |  |  |  |  |  |  |  |  |  |  |  |
| FP non-MH visit | 326 (97.0) | 3499 (96.2) | 5304 (95.3) | 1086 (93.2) | 1180 (96.0) | 1582 (97.3) | 1789 (96.2) | 2195 (95.6) | 3469 (94.0) | 8420 (95.8) | 1795 (93.7) |
| FP MH visit | 187 (56.2) | 1852 (50.9) | 2771 (49.8) | 473 (40.6) | 611 (49.7) | 779 (47.9) | 915 (49.2) | 1162 (50.6) | 1816 (49.2) | 4477 (49.0) | 806 (42.1) |
| Psychiatrist visit | 101 (30.3) | 762 (30.0) | 1006 (18.1) | 263 (22.6) | 226 (18.4) | 283 (17.4) | 361 (19.4) | 498 (21.7) | 764 (20.7) | 1942 (22.1) | 190 (9.92) |
| MH ED visit | 58 (17.4) | 476 (13.1) | 1110 (19.9) | 355 (30.5) | 169 (13.8) | 248 (15.2) | 328 (17.6) | 438 (19.1) | 816 (22.1) | 1567 (17.8) | 432 (22.6) |
| MH admission | 33 (9.9) | 227 (6.2) | 411 (7.4) | 160 (13.7) | 70 (5.7) | 108 (6.) | 119 (6.4) | 190 (8.3) | 344 (9.3) | 712 (8.10) | 119 (6.2) |
| ***Delivery to ED visit^d^*** |  |  |  |  |  |  |  |  |  |  |  |
| FP non-MH visit | 216 (64.9) | 2418 (66.5) | 3552 (63.8) | 714 (61.3) | 786 (64.0) | 1094 (67.3) | 1266 (68.1) | 1508 (65.7) | 2246 (60.8) | 5774 (65.7) | 1126 (58.8) |
| FP MH visit | 144 (43.2) | 1515 (41.6) | 2016 (36.2) | 344 (29.5) | 493 (40.1) | 642 (39.5) | 745 (40.0) | 852 (37.1) | 1287 (34.9) | 3414 (38.9) | 605 (31.6) |
| Psychiatrist visit | 57 (17.1) | 489 (13.4) | 518 (9.31) | 93 (7.98) | 135 (11.0) | 174 (10.7) | 190 (10.2) | 272 (11.8) | 386 (10.5) | 1079 (12.3) | 78 (4.07) |
| **Index ED Visit** |  |  |  |  |  |  |  |  |  |  |  |
| Days from delivery^e^ (Median, IQR) | 145 (50-253) | 148 (58-252) | 157 (68-256) | 150 (70-244) | 136 (57-250) | 145 (61-255) | 155 (63-257) | 158 (67-251) | 156 (68-254) | 166 (63-251) | 166 (72-262) |
| Self-harm | 14 (4.20) | 215 (5.9) | 392 (7.0) | 131 (11.2) | 72 (5.86) | 101 (6.21) | 123 (6.61) | 156 (6.79) | 300 (8.13) | 624 (7.10) | 128 (6.68) |
| Daytime visit | 141 (42.3) | 1629 (44.8) | 2245 (40.3) | 395 (33.9) | 549 (44.7) | 695 (42.7) | 789 (42.4) | 896 (39.0) | 1481 (40.1) | 3578 (40.7) | 832 (43.4) |
| Weekend visit | 97 (29.1) | 996 (27.4) | 1571 (28.2) | 336 (28.8) | 360 (29.3) | 461 (28.4) | 528 (28.4) | 627 (27.3) | 1024 (27.7) | 2466 (28.1) | 534 (27.9) |
| Academic hospital | 94 (28.2) | 836 (23.0) | 1099 (19.7) | 272 (23.4) | 249 (20.3) | 308 (18.9) | 358 (19.2) | 488 (21.2) | 898 (24.3) | 2172 (24.7) | 129 (6.7) |

^a^Related to most recent obstetrical delivery; ^b^Missing=17;  ^c^In 2 years prior to most recent obstetrical delivery; ^d^Between most recent obstetrical delivery and index ED visit ; ^e^Median (IQR)
SMM=Severe Maternal Morbidity; FP=Family Physician; MH=mental health; NICU=Neonatal intensive care unit; NR=Not reportable (all cells with <6 individuals or from which such cells could be calculated are suppressed as per ICES guidelines)

Table S2b. Additional baseline characteristics, presented by social determinant of health for immigration category, ethnicity, and ethnic diversity quintile.

|  | **Immigration category** | | | **Ethnicity** | | | **Ethnic Diversity Quintile (Q)^f^** | | | | |
| --- | --- | --- | --- | --- | --- | --- | --- | --- | --- | --- | --- |
|  | **Canadian-born/Long-term resident**  **(n=9,213)** | **Non-refugee immigrant**  **(n=1,176)** | **Refugee**  **(n=313)** | **Non-Chinese/ South Asian**  **(n=10,284)** | **Chinese**  **(n=183)** | **South Asian (n=235)** | **Q1**  **(Least Diverse) (n=3,691)** | **Q2**  **(n=2,296)** | **Q3**  **(n=1,860)** | **Q4**  **(n=1,626)** | **Q5**  **(Most Diverse)**  **(n=1,229)** |
| **Obsetrical^a^, Infant, and Medical** |  |  |  |  |  |  |  |  |  |  |  |
| Primiparous | 4353 (47.2) | 526 (44.7) | 121 (38.7) | 4793 (46.6) | 99 (54.1) | 108 (46.0) | 1041 (47.0) | 954 (45.5) | 950 (50.7) | 888 (49.3) | 1048 (44.8) |
| SMM ≥1 | 174 (1.9) | NR | NR | 200 (1.94) | NR | NR | 49 (2.2) | 45 (2.1) | 30 (1.6) | 35 (1.9) | 40 (1.7) |
| Preterm birth^b^ | NR | NR | NR | NR | NR | NR | NR | NR | NR | NR | NR |
| Multi-gestation | 170 | NR | NR | NR | NR | NR | NR | 33 (1.6) | 46 (2.4) | 38 (2.1) | 40 (1.7) |
| Stillbirth | 141 (1.5) | 17 (1.4) | 7 (2.2) | NR | NR | NR | 43 (1.9) | 26 (1.2) | 24 (1.3) | 21 (1.2) | 39 (1.7) |
| NICU admission | 1842 (20.7) | 148 (13.0) | 40 (13.3) | 1978 (19.9) | 11 (6.15) | 41 (18.1) | 420 (19.0) | 432 (20.6) | 377 (20.1) | 407 (22.6) | 458 (19.6) |
| Infant apprehension | 362 (4.1) | NR | NR | NR | NR | NR | 86 (3.9) | 84 (4.0) | 67 (3.6) | 66 (3.7) | 80 (3.4) |
| Infant death pre-ED | 107 (1.2) | 16 (1.4) | 7 (2.3) | NR | NR | NR | 29 (1.3) | 34 (1.6) | 18 (1.0) | 19 (1.0) | 35 (1.5) |
| Charlson score > 1 | 888 (9.6) | 69 (5.9) | 22 (7.0) | 43 (12.9) | 358 (9.8) | 499 (9.0) | 184 (8.3) | 190 (9.1) | 179 (9.6) | 172 (9.6) | 196 (8.4) |
| Asthma | 1314 (14.3) | 77 (6.6) | 25 (8.0) | 957 (9.31) | 7 (3.83) | 15 (6.38) | 309 (14.0) | 282 (13.4) | 272 (14.5) | 244 (13.6) | 277 (11.8) |
| Diabetes | 241 (2.6) | 40 (3.4) | 17 (5.4) | 1386 (13.5) | 9 (4.92) | 21 (8.94) | 42 (1.9) | 48 (2.3) | 60 (3.2) | 55 (3.0) | 79 (3.4) |
| Hypertension | 241 (2.6) | 50 (4.2) | 9 (2.9) | 280 | NR | NR | NR | 55 (2.6) | 39 (2.1) | 57 (3.2) | 92 (3.9) |
| **Prior Service Use** |  |  |  |  |  |  |  |  |  |  |  |
| ***Pre-delivery^c^*** |  |  |  |  |  |  |  |  |  |  |  |
| FP non-MH visit | 8768 (95.2) | NR | NR | 9805 (95.3) | NR | NR | 2073 (93.6) | 1996 (95.1) | 1790 (95.5) | 1735 (96.3) | 2282 (97.4) |
| FP MH visit | 4678 (50.8) | 476 (40.5) | 129 (41.2) | 5110 (24.7) | 57 (31.2) | 116 (49.4) | 1058 (47.8) | 1055 (50.3) | 972 (51.9) | 950 (52.8) | 1122 (47.9) |
| Psychiatrist visit | 1884 (20.4) | 196 (16.7) | 52 (16.6) | 2061 (20.0) | 29 (15.8) | 42 (17.9) | 347 (15.7) | 382 (18.2) | 423 (22.6) | 468 (26.0) | 497 (21.2) |
| MH ED visit | 1882 (20.4) | 94 (8.0) | 23 (7.4) | 1976 (19.2) | 7 (3.83) | 16 (6.81) | 477 (21.5) | 415 (19.8) | 366 (19.5) | 334 (18.6) | 302 (12.9) |
| MH admission | 756 (8.2) | 55 (4.7) | 20 (6.4) | 809 (7.87) | 10 (5.46) | 12 (5.11) | 165 (7.4) | 178 (8.5) | 151 (8.1) | 153 (8.5) | 158 (6.8) |
| ***Delivery to ED visit^d^*** |  |  |  |  |  |  |  |  |  |  |  |
| FP non-MH visit | 5788 (62.8) | 874 (74.3) | 238 (76.0) | 6599 (64.2) | 116 (63.4) | 185 (78.7) | 1335 (60.3) | 1350 (64.4) | 1164 (62.1) | 1183 (65.7) | 1687 (72.0) |
| FP MH visit | 3545 (38.5) | 386 (32.8) | 88 (28.1) | 3885 (37.8) | 53 (29.0) | 81 (34.5) | 788 (35.6) | 815 (38.8) | 740 (39.5) | 725 (40.3) | 862 (36.8) |
| Psychiatrist visit | 1013 (11.0) | 117 (9.95) | 27 (8.63) | 1113 (10.8) | 19 (10.4) | 25 (10.6) | 159 (7.2) | 229 (10.9) | 217 (11.6) | 264 (14.7) | 281 (12.0) |
| **Index ED Visit** |  |  |  |  |  |  |  |  |  |  |  |
| Days from delivery^e^ (Median, IQR) | 154 (65-254) | 147 (58-251) | 151 (66-244) | 154 (65-254) | 111 (42-227) | 127 (54-251) | 156 (66-252) | 161.5 (70-265) | 145 (62-243) | 142 (56-249) | 155 (64-248) |
| Self-harm | 592 (6.43) | 125 (10.6) | 35 (11.2) | 706 (6.87) | 19 (10.4) | 27 (11.5) | 115 (5.2) | 123 (5.9) | 121 (6.5) | 126 (7.0) | 214 (9.1) |
| Daytime visit | 3826 (41.5) | 471(40.0) | 113 (36.1) | 4251 (41.3) | 83 (45.4) | 76 (32.3) | 918 (41.4) | 887 (42.3) | 783 (42.8) | 759 (42.1) | 913 (39.0) |
| Weekend visit | 2577 (28.0) | 342 (29.1) | 81 (25.9) | 2883 (28.0) | 50 (27.3) | 67 (28.5) | 590 (26.6) | 584 (27.8) | 539 (28.8) | 529 (29.4) | 642 (27.4) |
| Academic hospital | 2002 (21.7) | 210 (17.9) | 224 (28.4) | 2239 (21.8) | 36 (19.7) | 26 (11.1) | 331 (14.9) | 399 (19.0) | 454 (24.2) | 593 (32.9) | 483 (20.6) |

^a^Related to most recent obstetrical delivery; ^b^Missing=17;  ^c^In 2 years prior to most recent obstetrical delivery; ^d^Between most recent obstetrical delivery and index ED visit ; ^e^Median (IQR); ^f^Missing=372; SMM=Severe Maternal Morbidity; FP=Family Physician; MH=mental health; NICU=Neonatal intensive care unit; NR=Not reportable (all cells with <6 individuals or from which such cells could be calculated are suppressed as per ICES guidelines)

Table S3. Risk of admission among postpartum individuals with psychiatric ED visits, present as N(%) admitted and relative risk adjusted for social determinants of health, severity, and all other variables in the table.

|  | **Admitted**  **N(%)** | **Fully-adjusted model**  **aRR (95% CI)** |
| --- | --- | --- |
| **Social Determinants of Health** |  |  |
| Age in years, 40 or older | 79 (23.7) | 1.00 (referent) |
| 30-39 years | 679 (18.7) | 0.91 (0.79-1.05) |
| 20-29 year | 802 (14.4) | 0.89 (0.76-1.05) |
| 19 years or under | 155 (13.3) | 0.90 (0.72-1.12) |
| Income Quintile, Q5 (highest) | 221 (18.0) | 1.00 (referent) |
| Q4 | 264 (16.2) | 0.96 (0.82-1.10) |
| Q3 | 296 (15.9) | 0.93 (0.80-1.08) |
| Q2 | 382 (16.6) | 0.92 (0.79-1.08) |
| Q1 (lowest) | 552 (15.0) | 0.90 (0.77-1.05) |
| Immigrant category, Canadian-born/Long-term resident | 1,380 (15.0) | 1.00 (referent) |
| Immigrant (non-refugee) | 269 (22.9) | 1.11 (0.98-1.26) |
| Refugee | 66 (21.1) | 1.05 (0.87-1.26) |
| Residence, Urban | 1,523 (17.3) | 1.00 (referent) |
| Rural | 192 (10.0) | 1.06 (0.90-1.26) |
| Ethnicity, Not Chinese or South Asian | 1,601 (15.6) | 1.00 (referent) |
| Chinese | 63 (34.4) | **1.49 (1.24-1.80)** |
| South Asian | 51 (21.7) | 1.05 (0.82-1.35) |
| Diversity, Q1 (least diverse) | 272 (12.3) | 1.00 (referent) |
| Q2 | 316 (15.1) | 1.07 (0.94-1.22) |
| Q3 | 329 (17.6) | 1.10 (0.96-1.25) |
| Q4 | 300 (16.7) | 0.92 (0.80-1.05) |
| Q5 (most diverse) | 473 (20.2) | 0.91 (0.79-1.03) |
| Missing | 25 (6.72) | 0.90 (0.79-1.03) |
| **Severity at Index ED visit** |  |  |
| Diagnosis, Anxiety | 134 (2.9) | 1.00 (referent) |
| Depressive | 760 (23.3) | **7.03 (5.87-8.44)** |
| Bipolar | 141 (54.9) | **13.12 (10.50-16.40*)*** |
| Psychotic | 335 (76.0) | **18.23 (15.09-21.01)** |
| Substance use | 70 (5.6) | **1.73 (1.26-2.39)** |
| Other | 275 (27.6) | **7.15 (5.81-8.81)** |
| CTAS Acuity, Low | 107 (4.7) | 1.00 (referent) |
| Moderate | 731 (13.2) | **2.35 (1.92-2.86)** |
| High | 877 (30.0) | **4.15 (3.32-5.19)** |
| **Obstetrical, Infant, and Medical^a^** |  |  |
| Primiparous (vs. multiparous) | 816 (16.3) | 0.96 (0.88-1.06) |
| Multi-gestation (vs. singleton) | 28 (14.0) | **0.72 (0.54-0.97)** |
| Severe Maternal Morbidity, ≥1 (vs. 0) | 36 (17.1) | 0.98 (0.75-1.29) |
| Preterm birth^b^ (vs. term) | 196 (16.0) | 1.01 (0.89-1.15) |
| Stillbirth (vs.^.^ liveborn) | 23 (13.9) | 1.19 (0.83-1.70) |
| NICU admission (vs. none) | 382 (17.8) | 1.10 (1.00-1.23) |
| Infant apprehension (vs. not) | 60 (15.3) | 0.90 (0.71-1.13) |
| Infant death pre-ED (vs. not) | 19 (13.2) | 1.28 (0.92-1.79) |
| Charlson score, ≥1 (vs. 0) | 164 (16.8) | 1.05 (0.92-1.20) |
| Asthma (vs. no asthma) | 189 (13.4) | 0.90 (0.79-1.02) |
| Diabetes (vs. no diabetes) | 50 (16.8) | 0.84 (0.67-1.06) |
| Hypertension (vs. no hypertension) | 44 (14.7) | 0.96 (0.76-1.23) |
| **Health Service Use Pre-delivery^c^** |  |  |
| Family physician non-MH visits, ≥1 (vs. 0) | 1,641 (16.1) | 0.96 (0.78-1.18) |
| Family physician MH visits, ≥1 (vs. 0) | 977 (18.5) | **1.15 (1.06-1.24)** |
| Psychiatrist visits, ≥1 (vs. 0) | 546 (25.6) | 1.05 (0.94-1.17) |
| MH ED visits, ≥1 (vs. 0) | 312 (15.6) | **0.89 (0.80-0.99)** |
| MH admissions, ≥1 (vs. 0) | 264 (31.8) | **1.35 (1.21-1.51)** |
| **Delivery to ED visit^d^** |  |  |
| Family physician non-MH visits, ≥1 (vs. 0) | 1,084 (15.7) | 0.99 (0.90-1.09) |
| Family physician MH visits, ≥1 (vs. 0) | 763 (19.0) | **1.16 (1.07-1.26)** |
| Psychiatrist visits, ≥1 (vs. 0) | 336 (29.0) | **1.12 (1.03-1.23)** |
| **Index ED Visit**^e^ |  |  |
| Days from delivery^f^ | 156 (67-257) | 1.0000 (0.9996-1.0004) |
| Evening/overnight visit (vs. daytime) | 1,015 (16.1) | **1.10 (1.02-1.19)** |
| Weekend visit (vs. weekday) | 423 (14.1) | 0.97 (0.89-1.06) |
| Community/small hospital (vs. academic/mental health/pediatric) | 414 (18.0) | 1.06 (0.90-1.25) |

^a^Obstetrical and infant variables are related to the most recent obstetrical delivery; ^b^Missing=17; ^c^In 2 years prior to most recent obstetrical delivery; ^d^Between most recent obstetrical delivery and index ED visit^; e^The variable representing self-harm in any diagnostic field was not included in the final model because it was collinear with diagnosis (VIF >2); ^f^Median (IQR); SMM=Severe Maternal Morbidity; FP=Family Physician; MH=mental health; NICU=Neonatal intensive care unit

Table S4a. Among those without primary substance use disorders, risk of admission within each social determinant of health; presented as crude risk, adjusted for clinical severity (diagnosis, acuity at triage, and comorbid substance use), and fully-adjusted for all covariates. Note that n(%) admitted is not included as it is not reportable for most variables due to potential for small cell reidentification.

| **Social determinant of health** | **Crude RR  (95% CI)** | **Severity-Adjusted RR (95% CI)** | **Fully-Adjusted^a^ RR (95% CI)** |
| --- | --- | --- | --- |
| **Age** **in years** |  |  |  |
| 40 or older | 1.00 (referent) | 1.00 (referent) | 1.00 (referent) |
| 30-39 years | **0.80 (0.67-0.97)** | 0.90 (0.78-1.04) | 0.93 (0.80-1.08) |
| 20-29 year | **0.68 (0.56-0.82)** | **0.84 (0.71-0.98)** | 0.91 (0.77-1.08) |
| 19 years or under | **0.66 (0.50-0.88)** | 0.85 (0.68-1.05) | 0.95 (0.76-1.18) |
| **Income Quintile** |  |  |  |
| Q5 (highest) | 1.00 (referent) | 1.00 (referent) | 1.00 (referent) |
| Q4 | 0.89 (0.73-1.08) | 0.96 (0.82-1.12) | 0.97 (0.84-1.13) |
| Q3 | 0.90 (0.73-1.10) | 0.93 (0.80-1.08) | 0.95 (0.82-1.10) |
| Q2 | 0.93 (0.74-1.15) | 0.92 (0.78-1.07) | 0.93 (0.79-1.09) |
| Q1 (lowest) | 0.86 (0.71-1.05) | 0.78 (0.76-1.01) | 0.92 (0.79-1.08) |
| **Immigrant Category** |  |  |  |
| Canadian-born/Long-term resident | 1.00 (referent) | 1.00 (referent) | 1.00 (referent) |
| Immigrant (non-refugee) | **1.21 (1.00-1.47)** | 1.07 (0.93-1.22) | 1.11 (0.98-1.27) |
| Refugee | 1.13 (0.81-1.58) | 0.99 (0.81-1.20) | 1.06 (0.88-1.28) |
| **Residence** |  |  |  |
| Urban | 1.00 (referent) | 1.00 (referent) | 1.00 (referent) |
| Rural | **0.75 (0.63-0.90)** | 1.02 (0.87-1.20) | 1.05 (0.89-1.24) |
| **Ethnicity** |  |  |  |
| Not Chinese or South Asian | 1.00 (referent) | 1.00 (referent) | 1.00 (referent) |
| Chinese | **1.81 (1.38-2.38)** | **1.45 (1.17-1.80)** | **1.49 (1.23-1.80)** |
| South Asian | 1.11 (0.82-1.50) | 1.03 (0.80-1.33) | 1.03 (0.80-1.32) |
| **Ethnic Diversity Quintile** |  |  |  |
| Q1 (least diverse) | 1.00 (referent) | 1.00 (referent) | 1.00 (referent) |
| Q2 | **1.22 (1.03-1.44)** | 1.11 (0.97-1.28) | 1.10 (0.96-1.26) |
| Q3 | **1.33 (1.14-1.54)** | 1.11 (0.98-1.25) | 1.12 (0.98-1.27) |
| Q4 | 1.15 (0.95-1.39) | 0.93 (0.80-1.08) | 0.91 (0.79-1.05) |
| Q5 (most diverse) | **1.28 (1.04-1.58)** | 0.95 (0.82-1.11) | 0.93 (0.81-1.06) |
| Missing | 0.65 (0.40-1.07) | 0.83 (0.56-1.24) | 0.89 (0.61-1.31) |

Table S4b. Risk of admission among those presenting without primary substance use disorders, adjusted for clinical severity, social determinants of health, and all other variables. Note that n(%) admitted is not presented as it is not reportable for most variables due to potential for small cell reidentification.

|  | **Fully-adjusted model**  **aRR (95% CI)** |
| --- | --- |
| **Social Determinants of Health** |  |
| Age in years, 40 or older | 1.00 (referent) |
| 30-39 years | 0.93 (0.80-1.08) |
| 20-29 year | 0.91 (0.77-1.08) |
| 19 years or under | 0.95 (0.76-1.18) |
| Income Quintile, Q5 (highest) | 1.00 (referent) |
| Q4 | 0.97 (0.84-1.13) |
| Q3 | 0.95 (0.82-1.10) |
| Q2 | 0.93 (0.79-1.09) |
| Q1 (lowest) | 0.92 (0.79-1.08) |
| Immigrant category, Canadian-born/Long-term resident | 1.00 (referent) |
| Immigrant (non-refugee) | 1.11 (0.98-1.27) |
| Refugee | 1.06 (0.88-1.28) |
| Residence, Urban | 1.00 (referent) |
| Rural | 1.05 (0.89-1.24) |
| Ethnicity, Not Chinese or South Asian | 1.00 (referent) |
| Chinese | **1.49 (1.23-1.80)** |
| South Asian | 1.03 (0.80-1.32) |
| Diversity, Q1 (least diverse) | 1.00 (referent) |
| Q2 | 1.10 (0.96-1.26) |
| Q3 | 1.12 (0.98-1.27) |
| Q4 | 0.91 (0.79-1.05) |
| Q5 (most diverse | 0.93 (0.81-1.06) |
| Missing | 0.89 (0.61-1.31) |
| **Severity at Index ED visit** |  |
| Diagnosis, Anxiety | 1.00 (referent) |
| Depressive | **7.08 (5.88-8.52)** |
| Bipolar | **13.40 (10.68-16.80)** |
| Psychotic | **18.37 (15.15-22.27)** |
| Other | **7.06 (5.71-8.74)** |
| Comorbid Substance Use (vs. none) | **1.42 (1.18-1.71)** |
| CTAS Acuity, Low | 1.00 (referent) |
| Moderate | **2.24 (1.83-2.75)** |
| High | **3.99 (3.18-5.00)** |
| **Obstetrical, Infant, and Medical^a^** |  |
| Primiparous (vs. multiparous) | 0.97 (0.88-1.07) |
| Multi-gestation (vs. singleton) | **0.75 (0.56-1.00)** |
| Severe Maternal Morbidity, ≥1 (vs. 0) | 1.03 (0.79-1.35) |
| Preterm birth^b^ (vs. term) | 1.02 (0.90-1.15) |
| Stillbirth ^(vs.^ liveborn) | 1.16 (0.81-1.67) |
| NICU admission (vs. none) | 1.10 (0.99-1.22) |
| Infant apprehension (vs. not) | 0.84 (0.67-1.06) |
| Infant death pre-ED (vs. not) | **1.42 (1.03-1.96)** |
| Charlson score, ≥1 (vs. 0) | 1.00 (0.87-1.16) |
| Asthma (vs. no asthma) | 0.89 (0.78-1.01) |
| Diabetes (vs. no diabetes) | 0.89 (0.78-1.01) |
| Hypertension (vs. no hypertension) | 0.93 (0.73-1.18) |
| **Health Service Use Pre-delivery^c^** |  |
| Family physician non-MH visits, ≥1 (vs. 0) | 0.92 (0.75-1.12) |
| Family physician MH visits, ≥1 (vs. 0) | **1.16 (1.06-1.26)** |
| Psychiatrist visits, ≥1 (vs. 0) | 1.07 (0.97-1.19) |
| MH ED visits, ≥1 (vs. 0) | **0.86 (0.77-0.95)** |
| MH admissions, ≥1 (vs. 0) | **1.30 (1.17-1.45)** |
| **Delivery to ED visit^d^** |  |
| Family physician non-MH visits, ≥1 (vs. 0) | 1.00 (0.90-1.10) |
| Family physician MH visits, ≥1 (vs. 0) | **1.13 (1.04-1.23)** |
| Psychiatrist visits, ≥1 (vs. 0) | **1.14 (1.04-1.25)** |
| **Index ED Visit**^e^ |  |
| Days from delivery^f^ | 1.0000 (0.9996-1.0005) |
| Evening/overnight visit (vs. daytime) | **1.11 (1.03-1.20)** |
| Weekend visit (vs. weekday) | 1.00 (0.92-1.09) |
| Community/small hospital (vs. academic/mental health/pediatric) | 1.05 (0.90-1.23) |

^a^Obstetrical and infant variables are related to the most recent obstetrical delivery; ^b^Missing=17; ^c^In 2 years prior to most recent obstetrical delivery; ^d^Between most recent obstetrical delivery and index ED visit^; e^The variable representing self-harm in any diagnostic field was not included in the final model because it was collinear with diagnosis (VIF >2); ^f^Median (IQR); SMM=Severe Maternal Morbidity; FP=Family Physician; MH=mental health; NICU=Neonatal intensive care unit
